# Auto-Intoxication*Read before the Central Texas Medical Society, Temple, Texas, July, 1907.

**Published:** 1908-07

**Authors:** R. B. Sellers

**Affiliations:** Comanche, Texas


### Original Articles.

For Texas Medical Journal.

### Auto-Intoxication.*

BY R. B. SELLERS, M. D., COMANCHE, TEXAS.

There are symptoms arising under certain conditions in the human organism which are indicative of a pathological change in the functions of some particular group of organs, which symptoms indicate a condition called auto-intoxication. Just what special organ or system is involved in producing this state, and the cause which brings it about has been a source of great interest to the leading physiologists and pathologists in their research work for a number of years. Whether this poison producing auto-intoxication is caused by some special bacteria or poison taken from the outside or is generated within the body through the decreased or abnormal secretions of some one organ or a group of organs is yet to be solved to the satisfaction of the entire medical world. Each side has its followers among some of the best men in the profession, and as each of us must reach our own conclusions through our own experiments and observation and especially what is given us by those who have gone deep into the research work, I shall make a condensed review of what I find of most interest on the subject.

In its strictest sense auto-intoxication means "self-empoisonment through the absorption of noxious products of katabolism." It is said by some to be caused by the decrease or absence of internal secretions of an important organ, as the spleen, pancreas, liver, the pituitary bodies or the adrenals. This refers chiefly to those forms of disease in which clinical and pathological experiments have shown the invariable changes in an organ of a secretory nature, as is found in Addison's disease, myxedema, exophthalmic goitre and many other diseases in which we find the normal functions of certain organs have been disturbed. The great majority of pathologists, both in this country and Europe, believe that Addison's disease is caused by a cessation of the functions of the adrenals, producing a general intoxication with a most remarkable group of symptoms as general adynamia, gastro-intestinal disturbances, marked nervous symptoms and a profound pigmentation of the entire body.

---

*Read before the Central Texas Medical Society, Temple, Texas, July, 1907.

There are several diseases that cause a discoloration of the skin, as malaria, pseudo-leukemia, pellagra, tuberculosis and carcinoma; in these there is no involvement of the adrenals, but a marked anemia which does not exist in Addison's disease, in which the blood count is normal. It is a disease of the suprarenal bodies in which the greater part of normal parenchyma has been destroyed.

In their normal state they are composed chiefly of parenchymatous cells which are well supplied with blood vessels and nerve elements, the medullated and nonmedullated, which have been traced to other organs, uniting with the semi-lunar ganglion and the other parts of the solar plexus, and also to the splanchnic, the vagus and phrenic nerves.

As these organs are secretory, they are essential to life, as was proven by Brown-Sequard, who says that all animals in which there was a complete removal of the adrenals died; and before death there was a discoloration of the skin like in Addison's disease, with all the symptoms of auto-intoxication. His statements were denied by many men for awhile, they saying that death did not follow the removal of the adrenals, but lately the experiments demonstrated by Brown-Sequard have been proven correct, that where there was a complete extirpation of the glands death would follow, while on the other hand, if any part of the adrenal was left, death did not occur, though there were symptoms of auto-intoxication.

It was also found that if the adrenals were completely removed, that the blood became toxic; in other words, it shows that there are substances in the circulation which under normal conditions are rendered harmless by the action of the adrenals, and that they became protective organs where toxic products are formed through the errors of metabolism.

The experiments of Jocobi have shown that there is an intimate relation between the adrenals and the peristaltic action of the intestines, and causes an increase of blood pressure, and gives a tonicity to the muscular walls.

A disturbance of these glands through the involvement of their parenchyma affects the secretion and produces a set of symptoms indicative of a general intoxication, as a marked disturbance of the gastro-intestinal system, the kidneys and the central nervous system, with continual pains in the epigastric, lumbar and occipital regions.

Diabetes is another condition from which auto-intoxication may develop. In those suffering from this malady the organs directly concerned in the absorption or the burning of sugar, have become damaged and are incapable of performing the work assigned them; that is, they fail to extract the sugar from the blood brought to them, and a glycosuria results, a defective metabolism, and for this reason sugar is excreted by the kidney. Conditions pass from bad to worse until the system becomes surcharged with the intoxicant, and the first outward manifestation we may have of the extreme auto-intoxication is when our patient develops diabetic coma. Quite a number of causes have been assigned for the development of diabetes, as injury to the fourth ventrical, brain tumors, infectious diseases, syphilis, epilepsy, heredity, obesity, and disease of the pancreas. Whatever be the origin, diabetes is a disease of malassimilation, and when the process of metabolism becomes greatly deranged, and glycosuria is marked, a new substance, oxybutyric acid, or a condition known as diabetic acidosis, is formed, which is very poisonous, and the only known cause of diabetic coma.

Another disease, myxedema, can be classed as resulting from an auto-intoxication. There is a disturbance of metabolism, of the circulation, and of the cerebral functions. The power of co-ordination is diminished and there is a loss of memory, hallucinations, drowsiness, convulsions and coma, all speak of the intoxication from which the patient is suffering. There is a general swelling of the body, and thickening of the skin to such an extent that our patient would scarcely be recognized. Albumen may be found in the urine in large amounts. In health the thyroid gland can be palpated, while in myxedema, Ewald says that in 80 per cent of cases the gland can not be felt. In 94.4 per cent of cases of myxedema, where autopsys were secured, the gland had changed into a shrunken remnant, a mass of hard, fibrous connective tissue which remarkably resembled the terminal stage of degenerative process so frequently met with in diseased kidneys and livers.

The experiments of Schiff on men show that a total extirpation of the thyroid with the supernumerary glands in all cases produce a myxedema, showing an involvement or interference with metabolism by the removal of this secretory organ, to be the cause of the auto-intoxication that invariably follows.

We will now speak of the relation of the pancreas and liver to disease. The terminal portion of the ductus choledochus is closely connected with the head of the pancreas, and that the vagus is the secretory nerve of this latter organ. Physiologists have become convinced that the pancreas is one of the most important organs of the body, and its functions play an important role in the process of digestion and metabolism, that of fat emulsifying and fat splitting power and eliminating sugar from the blood. The intimate relation of this organ to the liver, as we have said through the ductus choledochus, makes the liver a co-partner in all the diseases of metabolism wherein the pancreas blays a part.

The theory that jaundice is due to a disturbed function of the liver cells and is an auto-intoxication, is gaining ground every year.

Minkouski, of Cologne, says that the special function of the hepatic cell which enables it to introduce certain products into the bile channels, others into the blood vessels or lymph channels—biliary coloring matter and bile acids into the biliary passages and sugar and urea into the blood—is apparently dependent upon normal nutrition and normal function of the cell itself. He further says: "I compared the disturbance in function of the hepatic cells here described, with the disturbance in function of the renal cells which occur in albuminuria, and pointed to the analogous appearance of these forms of disturbance, in infectious diseases, in intoxications, in lesions of the nervous system, in circulatory disturbances and in the parenchymatous implication of the organs." He again says that besides a disturbance in the circulation of the blood, and a disturbance of the innervation of the liver, jaundice may come from the effects of poisons, as phosphorous and toxic bacterial products introduced with tainted foods (ptomains) or those formed in the organism in infectious diseases (toxins), as in pneumonia, septicemia, relapsing fever and yellow fever.

Another form of auto-intoxication that is of special interest is that of uremia. In acute or chronic nephritis there develops a condition in which the entire system becomes affected. From a pathological standpoint the kidneys are the principal organs involved, but the entire organism is dominated by a poison that has been generated in the body, where, we know not, but believing it to be a disease of metabolism, and as the kidneys are the organs most affected, we think it must be some action of the adrenals, through the circulation, on the glomeruli, causing the same destruction of the parenchyma which was observed by Minkouski in an analogous condition in the hepatic cells. This form of intoxication gives us a group of symptoms we know so well: first, a swelling of the lower extremities, then a gradual and sometimes a complete suppression of the urine, which is easily loaded with albumin, casts and blood. Our patient becomes drowsy, complaining of a bursting headache, increasing blindness, and then passes into convulsions and coma. There is no condition which comes on so suddenly and which causes so much alarm to the physician and so much danger to the patient as does eclampsia, which is a true auto-intoxication. Similar in many respects to the terminal stages of acute Bright's disease, it differs in this respect, that as soon as the child is delivered, the convulsions controlled and the bowels moved freely; if the patient be able to stand the strain for a few hours, all the poison is eliminated, albumin disappears, normal kidney conditions are soon re-established and the patient rapidly recovers. While this is an auto-intoxication, due no doubt to some defect of metabolism occurring only in pregnancy, and relieved as soon as that is terminated, yet in no other disease is there collected such an amount of so virulent a poison, which bursts with all the suddenness and fury of a cyclone.

There is a difference of opinion among pathologists as to auto-intoxication due, as some claim, to poisons generated by the absorption of foods or other foreign matter. Whether this condition, which is called ptomain poison, should be classed an auto-intoxication, is the question that disturbs many in our profession today. I hold that even if an article of diet, poisonous or not poisonous, when taken into the gastro-intestinal tract, causes a group of symptoms indicative of the generation of poisonous gases, that such condition is not a true auto-intoxication, but simply a ptomain poisoning; the distinction being this: in auto-intoxication there must be an abnormal condition of metabolism between certain systems or groups of organs, producing a set of symptoms of intoxication, but in no way connected with anything that may enter the system through the gastro-intestinal tract, while in ptomain or toxic poisoning the agent must be introduced from the outside. Still I think it best to speak to some extent of ptomain or toxic poison, giving the views why some hold this to be an auto-intoxication.

Under normal conditions there are bacteria in the intestines, and the greatest irritation to the intestinal wall is produced by bacterial toxins, and recognized as very poisonous; this condition is called ptomain poisoning and is brought about by tainted foods, as meats, fish, mushrooms and fruits, causing marked gastro-intestinal disturbances as nausea, vomiting, diarrhea, headache, abdominal pains, cold, clammy sweats, and in some cases, especially in old people, profound shock and death. That the system is suffering from an intoxication at this time can not be gainsaid, but whether the bacteria, per se, are responsible for this condition through the generation of a toxin from themselves, or they through their action upon the circulation, cause certain organs that take an active part in metabolism, to become involved and lessen their secretory function, is a matter to be determined by the continued research of the pathologist.

It has been a pertinent question for years, and there is none of greater interest, as to what part in the preservation of health does the blood play, as to its power to overcome bacterial disease?

There is in the blood an element called the phagocyte, a form of the leucocyte, which possesses the power of changing their shape, and have been called scavengers because of the ability with which they are credited of destroying any poison or substance, as bacteria, that may enter the circulation, and some physiologists contend that it is through their action that the system enjoys to so great an extent the immunity from disease. Sajous, perhaps the greatest authority on this theory in this country, says: "We have furnished evidence to the effect that the bacterial phagocytes, the neutrophiles, absorb trypsin, not only in the intestinal canal, but likewise in the portal vein; to this secretion we ascribe the destruction of all toxic albuminoids, toxins, vegetable poisons and venoms. Bacteria being likewise ingested, they are destroyed in the digestive vacuales of the phagocytic neutrophiles." He further says that there is a direct functional relation between the adrenal system and the leucocytes, "that the oxydizing substance which invades the blood stream in increased quantities under the influence of a toxic should have reached the structures mentioned at an early stage of the morbid process." We may then ask if the phagocytes possess such destructive power, why do not all diseases end favorably? The physiologists who believe that auto-intoxication is caused from substances taken into the system, as in ptomain and toxic poisons, as does Sajous, say that after the bacteria enter the body they give off a secretion, a product called toxins, which enter the circulation, and if it be a virulent form, as in tetanus and diphtheria, and if it is formed in large amounts, it overcomes or destroys the action of the phagocyte, and auto-intoxication develops and the patient dies.

The term auto-intoxication is used too frequently by physicians either through ignorance or to cover up the mistakes of diagnoses, they telling the family of a condition which is not understood by the laity and is also hazy to the physician.

There are many conditions arising in children in which there is an involvement of the gastro-intestinal tract, which is simply ptomain poisoning, and which can be relieved in the great majority of cases by judicious treatment in removing the poison from the system through the bowels and kidneys.

At the January meeting of the College of Physicians, Philadelphia, Dr. Forchimer, of Cincinnati, read a paper on "Chronic Intestinal Auto-intoxication," in which he said: "Strictly speaking, only that process should be called auto-intoxication which is caused by the toxic bodies resulting from metabolism, and not due to any exogenous cause, such as bacterial activity, and from the research of most of the prominent investigators, both in this country and Europe. We must consider that auto-intoxication is a result of defective metabolism and not in any way caused by bacterial infection."

